# Quarantine Laws

**Published:** 1845-09

**Authors:** 


### *Quarantine Laws.*

Extract from the Proceedings of the Physico-Medical Society of New Orleans.

At a regular meeting of the Physico-Medical Society of New Orleans, held Saturday evening, 15th February, 1845, an article read by Dr. Hort, on the subject of Quarantine laws, was, on motion of Dr. Farrell, referred to a committee of five, to report at a subsequent meeting. The President appointed on said committee Drs. Farrell, Hort, Jones, Anson and Dowler.

At the regular meeting of the same Society, Saturday evening, May 10th, the following report was submitted by the committee and unanimously adopted.

The committee appointed to report on the expediency of Quarantine laws as a means of preventing the importation of yellow fever into this city, beg leave to state, that in considering this subject they have felt the responsibility which appertains to questions affecting the health and prosperity of the city, and the lives of the inhabitants.

That while they admire and appreciate the ability with which several eminent medical men have advocated the contagiousness of yellow fever, its importation from the Eastern into the Western hemisphere, and the consequent necessity of the establishment of Quarantine laws, they nevertheless consider that the weight of testimony and of facts is immeasurably on the other side of the question; and which opinion is further confirmed by their own experience and observation.

That they can see no reason why the same local and general causes, under the same circumstances, or very nearly so, should not produce similar results in the production of malignant fevers, in both hemispheres of the world.

That where sufficient causes exist to engender disease in one place, it is useless to speculate on the question of its importation from some other place.

That in reviewing the history of yellow fever for one hundred and fifty years past, the committee have come to the conclusion that it was developed, as were many other malignant diseases, before unknown, by the march of civilization urged forward by commercial enterprise.

That in this way, in the course of time, yellow fever became developed in both hemispheres, confined within nearly the same parallels of latitude, and forming distinct yellow fever regions, in addition to the regions of cholera and plague.

That in the gradual progress of civilization, measures have been adopted, and changes of climate have taken place, which have greatly diminished the yellow fever region in this hemisphere; and that its northern limit is now twelve degrees south of what it was a hundred years ago, in the time of Lind.

That this great result has been accomplished, not by quarantine laws, but by other judicious police regulations, together with great changes in the local features of countries; and those atmospherical changes, over which man has no control.

That quarantine laws, even should their existence be deemed necessary, are inadequate to the protection of a seaport of easy access ; as Dr. Rush says, that a *still more rigid* quarantine called for in 1797, in Philadelphia, failed to accomplish the purpose desired. In 1805, the same fact is affirmed by Dr. Rogers, health officer at New York. In 1842, if imported, this system again failed at New York, (and in this city, it signally failed in 1820 or '21, when a rigid quarantine was established at the English turn.)

The committee are therefore of opinion, that quarantine laws are unnecessary and inexpedient for the protection of the city.

That even if they did prevent the importation of yellow fever (admitting for one moment, for argument sake, that the disease might be imported), they could not at any rate prevent the existence of diseases equally fatal; such as the congestive fever, and the malignant types of intermitting and remitting fevers.

That facts seem clearly to prove, that the yellow fever has decreased in malignity, in a ratio with the improvements of the city—as the draining of the land in the rear of the city ; the paving of the streets ; the filling up of empty lots ; the use of asphaltum ; permitting the river water to run through the streets, when the river is high ; and the removal of filth and offal from the streets.

That instead of quarantine laws, the measures last alluded to should be steadily persevered in, and carried, by an enlightened policy, to a still greater extent ; which would not only have a tendency to avert yellow fever, but all other malignant diseases, peculiar to our climate and position, at a particular season of the year.

The committee, in conclusion, sum up this report by declaring :

That they believe the yellow fever to be a disease of local or domestic origin, and that it is not an imported disease.

That it is never contagious.

That it may be made to yield to judicious police regulations.

That quarantine laws are very expensive to the community, and that they are not only unnecessary and inexpedient, but worse than useless. They therefore recommend :

1. That the commissaries in each ward be required to look into back yards and lots ; and be authorized to cause everything offensive to be promptly removed.

2. That the different Counsels of the city should exert themselves to the utmost in their official capacity, to have the surface of the earth covered over with something, to prevent the exhalations from the alluvial soil on which the city is built ; either round or paving stones, or bricks, or shells and sand, or asphaltum.

3. That the owners should be compelled by law to fill up all low swampy lots within the limits of the city.

4. That all offal deposited in the streets should be promptly removed ; and, if possible, before the heat of the day.

5. That whenever the river is high, the water should be allowed to run through the streets day and night : and that when it is too low, the water works, or if necessary, additional works established for the purpose, should be brought into play.

6. That above all, particular attention should be paid by the city authorities, to the alluvial bank, particularly under the wharves of the Second Municipality, which is annually uncovered as the river falls, exposing an immense surface of fresh deposit, covered with every kind of decaying vegetable and animal matter, which daily accumulates, either carried there by eddy currents of the river, or thrown in by the inhabitants.

The committee deem this last consideration to be of the highest importance, as there is every reason to believe that the bank of the river, under the wharves, is more productive of disease in the summer than all other causes in the city, combined.

7. That instead of depositing the filth and offal collected in the streets by the scavengers, in empty lots or in the rear of the city, it is recommended to the city authorities to have all such filth and offal thrown into the current of the river.

They would also observe, that the measures just recommended would not be attended with one-fourth of the expense of a quarantine establishment properly conducted; while, should they be pushed forward with zeal and energy, the time might, and no doubt would, ere long arrive, when New Orleans would no longer be within the yellow fever region; and consequently exempt, not only from that pestilence, but from all the other fatal diseases of the summer and fall, peculiar to our climate and to our position. This accomplished—what would there remain to retard the growth and prosperity of our city? She would speedily accomplish her high destiny, and in less than a quarter of a century become the most wealthy, prosperous, and populous city in the western hemisphere.—*New Orleans Med. Journal.*

